# Estrogen to Progesterone Ratio and Fluid Regulatory Responses to Varying Degrees and Methods of Dehydration

**DOI:** 10.3389/fspor.2021.722305

**Published:** 2021-10-14

**Authors:** Gabrielle E. W. Giersch, Nisha Charkoudian, Margaret C. Morrissey, Cody R. Butler, Abigail T. Colburn, Aaron R. Caldwell, Stavros A. Kavouras, Douglas J. Casa

**Affiliations:** ^1^Thermal and Mountain Medicine Division, United States Army Research Institute for Environmental Medicine, Natick, MA, United States; ^2^Oak Ridge Institute for Science and Education, Belcamp, MD, United States; ^3^Korey Stringer Institute, Department of Kinesiology, University of Connecticut, Storrs, CT, United States; ^4^Hydration Science Laboratory, Arizona State University, Tempe, AZ, United States

**Keywords:** female sex hormones, fluid balance, fluid restriction, copeptin, exercise heat stress

## Abstract

The purpose of this study was to investigate the relationship between volume regulatory biomarkers and the estrogen to progesterone ratio (E:P) prior to and following varying methods and degrees of dehydration. Ten women (20 ± 1 year, 56.98 ± 7.25 kg, 164 ± 6 cm, 39.59 ± 2.96 mL•kg•min^−1^) completed four intermittent exercise trials (1.5 h, 33.8 ± 1.3°C, 49.5 ± 4.3% relative humidity). Testing took place in two hydration conditions, dehydrated via 24-h fluid restriction (Dehy, USG > 1.020) and euhydrated (Euhy, USG ≤ 1.020), and in two phases of the menstrual cycle, the late follicular phase (days 10–13) and midluteal phase (days 18–22). Change in body mass (%BMΔ), serum copeptin concentration, and plasma osmolality (P_osm_) were assessed before and after both dehydration stimuli (24-h fluid restriction and exercise heat stress). Serum estrogen and progesterone were analyzed pre-exercise only. Estrogen concentration did not differ between phases or hydration conditions. Progesterone was significantly elevated in luteal compared to follicular in both hydration conditions (Dehy—follicular: 1.156 ± 0.31, luteal: 5.190 ± 1.56 ng•mL^−1^, *P* < 0.05; Euhy—follicular: 0.915 ± 0.18, luteal: 4.498 ± 1.38 ng·mL^−1^, *P* < 0.05). As expected, E:P was significantly greater in the follicular phase compared to luteal in both hydration conditions (Dehy—F:138.94 ± 89.59, L: 64.22 ± 84.55, *P* < 0.01; Euhy—F:158.13 ± 70.15, L: 50.98 ± 39.69, *P* < 0.01, [all •10^3^]). Copeptin concentration was increased following 24-h fluid restriction and exercise heat stress (mean change: 18 ± 9.4, *P* < 0.01). We observed a possible relationship of lower E:P and higher copeptin concentration following 24-h fluid restriction (*r* = −0.35, *P* = 0.054). While these results did not reach the level of statistical significance, these data suggest that the differing E:P ratio may alter fluid volume regulation during low levels of dehydration but have no apparent impact after dehydrating exercise in the heat.

## Introduction

It is well-established that dehydration can have marked effects on cognition, physiology, and performance. For research purposes, dehydration is often elicited through a variety of methods, including water restriction, passive heating and exercise stimuli. Each method elicits different volume regulatory responses and body mass loss (Stachenfeld and Keefe, 2002; Stacey et al., 2018a; Giersch et al., 2020), and can induce different outcomes. Dehydration via fluid restriction can lead to body mass losses of ~1% (Giersch et al., 2020), while dehydrating exercise in the heat can yield body mass deficits anywhere from 1–3% (Sawka et al., 2001). During mild dehydration (<2% body mass loss), mood has been shown to be negatively impacted (Ganio et al., 2011; Armstrong et al., 2012; Suh et al., 2018), while higher levels of body mass loss (>2%) result in impaired cognition (Wittbrodt and Millard-Stafford, 2018) and decreased endurance performance (Goulet, 2011). However, these data have been largely collected in men.

Understanding the mechanisms that may contribute to the dehydration response is helpful when making recommendations for fluid needs (Perrier, 2017). Mechanistically, fluid regulatory responses, such as arginine vasopressin (AVP), a primary fluid regulatory hormone, and copeptin, a surrogate marker for AVP (Morgenthaler et al., 2006), are commonly increased by dehydration stimuli (Stacey et al., 2018a; Popovic et al., 2019; Giersch et al., 2020; Suh et al., 2021). Specifically, copeptin concentration is increased with fluid restriction (Giersch et al., 2020), hypertonic saline infusion (Suh et al., 2021), and exercise (Popovic et al., 2019). Changes in fluid regulatory hormones can alter body fluid balance, increasing fluid retention with increased AVP concentration.

For women, sex hormones interact with the mechanisms governing body fluid balance and circulating levels of fluid regulatory hormones are altered by changes in estrogen and progesterone concentration (Stachenfeld et al., 2000; Calzone et al., 2001; Stachenfeld and Taylor, 2005; Stachenfeld, 2008; Giersch et al., 2019). Estrogen is positively related to AVP and high estrogen concentration decreases the osmotic threshold for AVP synthesis (Stachenfeld et al., 1999; Stachenfeld and Keefe, 2002), suggesting increased AVP secretion at lower levels of dehydration. Estrogen has also been positively correlated with copeptin across the menstrual cycle (Blum et al., 2014). Progesterone has also been observed to impact fluid retention via aldosterone and AVP pathways, and may also increase plasma volume independent of estrogen (Calzone et al., 2001; Stachenfeld and Taylor, 2005), but the precise mechanism of action of progesterone in this regard remains unclear. While estrogen and progesterone both have independent functions throughout the body, they appear to have opposing effects with respect to vascular function (Stephenson and Kolka, 1999; Wenner et al., 2011) and body temperature regulation (Stachenfeld et al., 2001a; Charkoudian and Stachenfeld, 2014). This relationship may also be present with respect to body fluid balance given the varying mechanisms through which estrogen and progesterone alter fluid regulation (Calzone et al., 2001; Stachenfeld et al., 2001b; Stachenfeld and Keefe, 2002; Stachenfeld and Taylor, 2005). Thus, fluid volume regulation may be altered based the concentrations of the hormones relative to each other at a given point in the menstrual cycle (Owen, 1975). This highlights the importance of assessing the relationship between estrogen and progesterone, or the estrogen-to-progesterone ratio (E:P). E:P incorporates the relative concentrations of the two hormones in circulation, allowing for consideration of which is dominant at any given time.

Understanding the possible role that estrogen, progesterone, and E:P may play in body fluid regulation during dehydration can aid in the understanding of fluid regulatory mechanisms and inform future research and recommendations for fluid needs in women. The purpose of this study was to perform an exploratory analysis to investigate the relationship between volume regulatory biomarkers, progesterone, estrogen, and E:P ratio, in menstrual cycle phases where estrogen is elevated, following varying degrees of dehydration elicited through fluid restriction and exercise heat stress.

## Methodology

### Ethical Approval

All research participants provided written informed consent. All study protocols and procedures were approved by the University of Connecticut Institutional Review Board under protocol nos. (H18–220 and H19–049). All data were collected in accordance with the Declaration of Helsinki. These data were collected as part of a larger investigation in which alternative aspects have previously been described (Giersch et al., 2020, 2021). Given the exploratory nature of this analysis as a part of a larger investigation, an *a priori* power calculation was conducted for alternative aspects, and thus was not conducted for the variables of interest described here.

### Subjects

Thirteen women were initially screened and enrolled in this study. Two women voluntarily withdrew from the study based on time commitment. One woman completed all aspects of the study, but her data were removed *post-hoc* following sex hormone analysis outside of expected ranges (Owen, 1975), as well as outside two standard deviations from the group mean within each phase. Thus, 10 women are included in these analyses. All participants were screened by a physician for contraindications to 24-h fluid restriction and exercise in the heat and were confirmed to have no chronic illnesses or medications that would influence fluid balance or responses to exercise heat stress. An internally developed menstrual history questionnaire was collected from all participants for scheduling of trials in appropriate phases, and to verify eumenorrhea (25–35 day cycles) without oral contraceptive use within six months. All participants were instructed to refrain from vigorous exercise and alcohol for 24 h prior to any visit to the laboratory, as well as caffeine consumption for 12 h before all exercise trials.

### Procedures

This investigation consisted of four exercise trials in a block randomized and counterbalanced order, separated by at least 72 h. Two exercise trials were conducted in each menstrual cycle (one in each phase), one euhydrated elicited via *ad libitum* fluid consumption (urine specific gravity, USG ≤ 1.020, Euhy), or dehydrated, elicited via 24-h fluid prescription (USG > 1.020, Dehy). Participants were provided instruction on how to achieve euhydration via *ad libitum* consumption during familiarization visits. Specifically, if participants arrived for a familiarization visit with a USG > 1.020, they were instructed to consume an additional 500 mL of fluid with dinner the evening before the next visit to ensure euhydration. Exercise trials were scheduled based on self-reported menstrual history, then verified *post-hoc* (by hormone concentration) for the late follicular (days 10–13) and midluteal (days 18–22) phases of the menstrual cycle. These specific menstrual phases were chosen in order to compare the impact of elevations in estrogen alone, vs. elevated estrogen + progesterone on body fluid balance during dehydration. Testing was conducted over two to three menstrual cycles to ensure that participants completed one trial within the reported day ranges for each phase over the course of two to three menstrual cycles, based on block randomization. All trials were conducted in the morning hours between 0600 and 1100, with each individual participant starting all trials within one hour of previous start times. Participants arrived at the laboratory 24-h before their scheduled trial to collect baseline hydration and body mass to calculate body mass change (%BMΔ) and receive their fluid prescription (euhydrated or dehydrated). Upon arrival for exercise trials, participants provided a morning urine sample to confirm hydration status, nude body mass, and pre-trial blood samples. Immediately following blood sampling, participants were provided 200 mL of fluid and then entered the environmental chamber. Participants were seated in the chamber for 15 min and then began the exercise trial. Exercise intensities during the trial were prescribed based on heat production as previously described (Cramer and Jay, 2019). Exercise trials consisted of three 45-min blocks broken down into 15 min of treadmill jogging (between 7 and11 W•kg^−1^), 15 min of walking (between 5 and 7 W•kg^−1^), and 15 min of seated rest in the heat (33.8 ± 1.3°C, 49.5 ± 4.3% relative humidity). No fluid was provided to participants during exercise. Following exercise, participants had a blood sample taken and provided nude body mass.

### Measurements

Prior to participation in exercise trials, participants completed a VO_2max_ test using a treadmill running incremental protocol, with speed increasing every three min until participants volitional exhaustion. Expired air was collected continuously throughout the test and was analyzed using indirect calorimetry (TrueOne 2400, ParvoMedics, Salt Lake City, UT, USA). Criteria used to determine VO_2max_ were rating of perceived exertion >17, respiratory exchange ratio >1.1, and ±10 beats per min of age-predicted heart rate max. Height was also assessed at this visit using a wall-mounted stadiometer (Seca, model 220, Hamburg, Germany). Body mass was assessed at each visit (Ohaus Defender 3000, Ohaus Corporation, Parisppany, NJ, USA). Pre- and post-exercise blood samples were analyzed for serum copeptin concentration and plasma osmolality (P_osm_). After blood was collected from an antecubital vein, serum samples were permitted to clot for 30–90 min at room temperature and then centrifuged (Eppendorf 5810R, Eppendorf AG, Hamburg, Germany; 4°C) at 3,000 rpm for 15 min, aliquoted and stored frozen (−80°C) for analyses. Plasma samples, via lithium heparin, were immediately centrifuged at 3,000 rpm for 15 min and analyzed for P_osm_ using freezing point depression osmometry following manufacturer guidelines (OsmoPRO; Advanced Instruments, Norwood, MA). Serum copeptin concentration was analyzed from single samples using immunofluorescent Copeptin proAVP KRYPTOR assay via the fully automated random-access immunoassay KRYPTOR Compact PLUS analyzer (BRAHMS, Thermo Fisher, Hennigsdort, Germany). Female sex hormones were measured from pre-exercise blood samples only and analyzed via enzyme-linked immunoassay in duplicate following manufacturer recommendations (17 ß-estradiol, sensitivity 10 pg•mL^−1^ and progesterone, sensitivity 0.1 ng•mL^−1^; ALPCo Diagnostics, Salem, NH). Any coefficient of variation (CV) >15% between samples was rerun for reliability, with all samples included in calculation of inter- and intra-assay CVs. E:P ratio was calculated by converting estrogen to from pg•mL^−1^ to ng•mL^−1^, divided by progesterone. This ratio is reported in •10^3^. Body mass change (%BMΔ) was calculated from nude body mass at baseline, after 24-h fluid restriction, and exercise trials including the consumption of 200 mL of fluid prior to exercise.

### Data Analysis

All analyses were conducted in R (R version 4.0.2) and SPSS v27 (IBM, Armonk, NY). Due to skew in the data, it was determined that assumption of normality was not tenable for some measures. Therefore, power transformations were applied to progesterone and the E:P ratio (0.2 and −0.2, respectively) following a Box-Cox analysis. Similarly, a log transformation was applied to the copeptin data after it was determined this best normalized the residuals compared to other transformations. Data are presented as mean ± standard deviation and significance was set *a priori* at *P* < 0.05. Linear mixed effects models were utilized to assess the influences of estrogen, progesterone, and E:P on fluid regulatory responses to fluid restriction and dehydrating exercise in the heat (Bates et al., 2014; Lenth, 2021). Correlation coefficients accounting for the multi-level nature of the data (many observations within each participant) were calculated with the “correlation” R package (Makowski et al., 2020).

## Results

Ten women completed all aspects of the investigation (age: 20 ± 1 year, weight: 56.98 ± 7.25 kg, height: 164 ± 6 cm, VO_2max_: 39.59 ± 2.96 mL•kg•min^−1^); baseline hydration measures can be found in Table 1. As expected, progesterone concentrations were higher in the midluteal phase compared to the late follicular phase in both hydration conditions (Dehy—follicular: 1.156 ± 0.31 ng·mL^−1^, luteal: 5.190 ± 1.56 ng•mL^−1^, *P* < 0.05; Euhy—follicular: 0.915 ± 0.18 ng·mL^−1^, luteal: 4.498 ± 1.38 ng·mL^−1^, *P* < 0.05, inter-assay *CV* = 1.39, intra-assay *CV* = 2.35). The E:P ratio was higher in the late follicular phase compared to the midluteal phase in both hydration conditions (Dehy—follicular:138.94 ± 89.59, luteal: 64.22 ± 84.55, *P* < 0.01; Euhy—follicular:158.13 ± 70.15, luteal: 50.98 ± 39.69, *P* < 0.01, all •10^3^) with no E:P ratio <1. There were no differences in estrogen concentration between phases (Dehy—follicular: 112.33 ± 46.47 pg•mL^−1^, luteal: 130.82 ± 67.15 pg•mL^−1^, *P* > 0.05; Euhy—follicular: 112.40 ± 52.35 pg•mL^−1^, luteal: 121.48 ± 77.40 pg•mL^−1^, *P* > 0.05, inter-assay *CV* = 1.59, intra-assay *CV* = 8.30).

**Table 1:** Pre-exercise USG and %BMΔ observed between dehydrating stimuli.

	**Follicular Dehy**	**Luteal Dehy**	**Follicular Euhy**	**Luteal Euhy**
Pre-exercise USG	1.013 ± 0.009	1.019 ± 0.006	1.018 ± 0.008	1.016 ± 0.011
Pre-exercise %BMΔ	−1.15 ± 0.85	−0.74 ± 0.41	0.31 ± 1.63	0.25 ± 1.28
Exercise dehydration %BMΔ	−1.76 ± 0.64	−2.26 ± 0.59	−2.42 ± 1.21	−2.28 ± 0.54
Total %BMΔ	−2.91 ± 1.22	−2.98 ± 0.76	−2.13 ± 1.77	−2.03 ± 1.63

*Data presented as mean ± standard deviation*.

**Figure 1:**
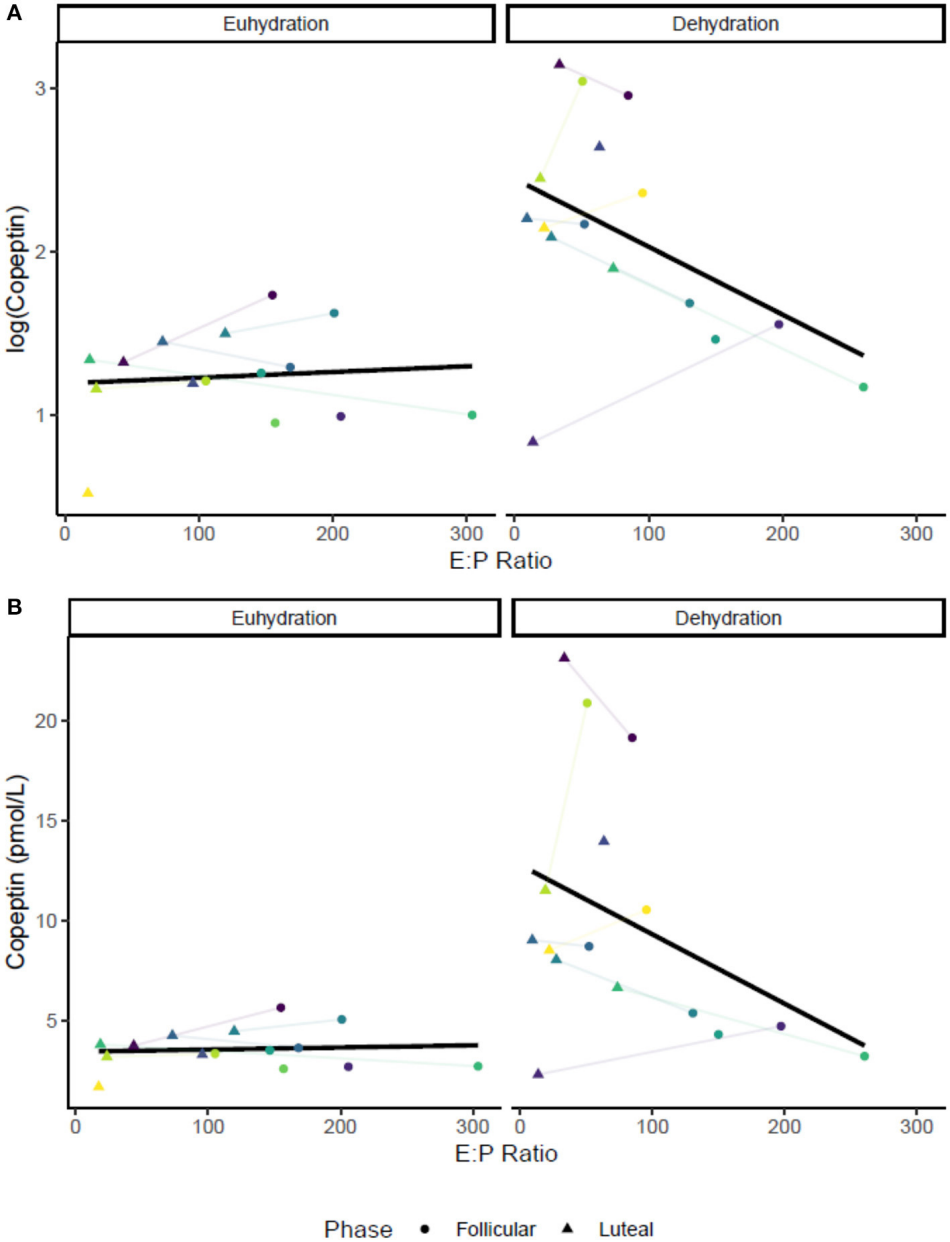
Relationships between E:P [all •10^3^] and pre-exercise copeptin concentration in both conditions: euhydrated (*ad libitum* fluid consumption for 24-h, USG ≤ 1.020, *P* > 0.05) and dehydrated (24-h fluid restriction, USG > 1.020, *P* = 0.054). Colored dots show individual data for in both trials (follicular and luteal). **(A)** shows log-transformed copeptin data, and **(B)** shows raw values. Colored dots show individual trials; lines between dots connect individual trials within participants.

No relationship was apparent between pre-exercise copeptin concentration and estrogen (*r* = −0.12, *P* = 0.51) or progesterone (*r* = −0.22, *P* = 0.24). Copeptin and P_osm_ were also increased by dehydration via exercise in the heat without any apparent influence of estrogen, progesterone, or E:P (copeptin—mean change: 18 ± 9.4, *P* < 0.01; P_osm_–mean change: 4.4 ± 3.97, *P* < 0.01). Post-exercise plasma osmolality and post-exercise copeptin concentration were significantly correlated (*r* = 0.60, *P* < 0.01) without any apparent impact of sex hormones or initial hydration status. Figure 1A shows the relationship between the log transformed copeptin data and E:P, Figure 1B shows the relationship between raw (untransformed) copeptin values and E:P, with colored dots showing individual responses across two trials (follicular and luteal) in each hydration condition; follicular phase trials are found in the colored circles, while luteal phase trials are found in the colored triangles, lines between shapes connect individual trials within the same subject. Varying degrees of %BMΔ were observed with no effect of menstrual cycle phase as shown in Table 1 (*P* > 0.05*)*. Copeptin concentration was increased by 24-h fluid restriction in both groups with a possible influence of E:P ratio (Figure 1), but this relationship did not reach the level of statistical significance (*P* = 0.054).

## Discussion

The purpose of this investigation was to assess the impact of estrogen, progesterone, and E:P on body fluid regulatory responses following varying degrees and methods of dehydration in menstrual cycle phases where estrogen concentrations are elevated. The relationships among copeptin concentration, estrogen, progesterone, and E:P following dehydrating exercise in the heat (greater dehydration) were small and inconsistent. We show a possible novel effect of E:P at lower levels of dehydration (body mass loss ~-1%), where lower E:P (i.e., increased estrogen and progesterone together) was associated with higher copeptin concentrations. While the relationship between E:P and pre-exercise copeptin was not statistically significant (*P* = 0.054), the confidence interval included rather large effects (r = −0.34 95% C.I. [−0.63, 0.01]). Estrogen and progesterone individually did not appear to have consistent influences on any pre-exercise measures, suggesting utility of E:P as a possible influential marker of fluid balance in addition to estrogen and progesterone. It is possible that E:P was more impactful than either estrogen or progesterone independently possibly due to the opposing actions of the individual sex hormones, particularly with respect to fluid balance (Calzone et al., 2001; Stachenfeld et al., 2001b; Stachenfeld and Keefe, 2002; Stachenfeld and Taylor, 2005).

To our knowledge, this is the first investigation to assess the relationship of E:P ratio and fluid regulatory hormones in the late follicular and midluteal phases following passive dehydration via fluid restriction and exercise heat stress. Previously, Stachenfeld and Keefe (2002), assessed the impact of estrogen alone and estrogen with progesterone on responses to hypertonic saline infusion. Elevated estrogen levels appeared to increase fluid retention, whereas estrogen and progesterone together appeared to also increase sodium retention. Progesterone alone does not appear to alter threshold for AVP secretion like estrogen following hypertonic saline infusion (Calzone et al., 2001), although those mechanisms remain unclear (Stachenfeld et al., 2001b). Our results do not show any impact of high estrogen with or without high progesterone on copeptin response, but the relationship between E:P ratio and copeptin indicates there may be an effect and warrants further investigation into the possible role of progesterone (Calzone et al., 2001; Stachenfeld et al., 2001b; Stachenfeld and Keefe, 2002; Stachenfeld and Taylor, 2005). From a practical perspective, it would appear that the normal hormonal variation that occurs across the menstrual cycle does not alter body mass loss from dehydration due to exercise in the heat.

Our data are consistent with the idea that copeptin or AVP may be a key regulator at low levels of dehydration but may be less impactful when responding to a larger dehydration stimulus (Melin et al., 1997). Since AVP acts primarily on the kidney to promote fluid retention through the kidney tubules (Jaenike and Waterhouse, 1961), with greater levels of body mass loss, less fluid is available for retention and increased fluid consumption is required to recover these losses. The level of %BMΔ observed in this investigation following exercise heat stress would likely negatively impact cognition and athletic endurance performance based on recent meta-analyses (Goulet, 2013; Wittbrodt and Millard-Stafford, 2018), despite the relatively low level and no differences between groups. Based on our present data for fluid regulatory responses and body mass loss, it appears women do not require different fluid replacement following exercise heat stress based on menstrual cycle phase or sex hormone concentration.

The observed increase in copeptin concentration following exercise in the heat is consistent with previously reported results in men (Popovic et al., 2019), suggesting women have a similar fluid regulatory response to dehydrating exercise. The exercise copeptin response did not appear to be moderated by sex hormone concentration or E:P. While estrogen has been observed to be related to copeptin concentration at rest (Blum et al., 2014), it does not appear that the relationship is affected or apparent with integration of dehydrating stimuli. It is possible that the effect of exercise and dehydration (>2% body mass loss) on fluid regulation is large enough to wash out any effect of estrogen, progesterone, or their ratio may have. It's also valuable to note that greater levels of dehydration elicited passively may elicit different responses than the exercise heat stress stimulus used in this investigation. Exercise heat stress independently stimulates AVP pathways, so results in this investigation are specific to dehydration resulting from exercise heat stress. Our results are consistent with previous literature showing exercise stimulates AVP pathways independently (Stacey et al., 2018a,b).

The potential influence of lower E:P ratio on increased copeptin at lower levels of dehydration (i.e., before exercise) may suggest a possible practical impact of E:P on fluid regulation at low levels of dehydration, similar to those commonly associated with activities of daily living (i.e., changes in hydration corresponding to ~1% or less of body mass). This is particularly true given the number of women who may not consume adequate amounts of fluid daily (Gibson and Shirreffs, 2013). AVP concentration is responsive to changes in behavioral fluid consumption and appears to be the primary fluid regulator in women who are both high and low fluid consumers when reproductive hormones were low (Johnson et al., 2016). In this context, behavioral fluid consumption was not assessed in our present investigation and warrants future investigation with respect to the possible impact of sex hormones.

### Strengths and Limitations

This investigation utilized a block randomized design where participants completed all trials, serving as their own controls to assess the possible influence of estrogen, progesterone, and the E:P ratio on fluid balance following varying degrees/methods of dehydration. Limitations include the fact that, as a *post-hoc*, exploratory analysis, no *a priori* power calculation was conducted to ensure appropriate statistical power. Accurately predicting hormonal variations is difficult, and the estrogen and progesterone concentrations that we report in this study are not consistent with those expected in a typical late follicular phase and ovulatory midluteal phase, respectively (Owen, 1975). In this context, it is likely that the peaks within each phase were missed in scheduling. It is possible that higher progesterone would have a different effect on the E:P ratio and the possible influence of E:P on fluid regulation. Although we did not collect 24-h morning samples, first morning samples is a validated method to assess starting hydration status in these participants (Pepper, 1924; Armstrong et al., 1994). Diet and fluid records and behavioral fluid consumption were not assessed to quantify total water intake, which may have impacted our findings, particularly with respect to copeptin concentration (Johnson et al., 2016).

## Conclusions

We show a possible novel utility of E:P as a measure in addition to individual concentrations of estrogen and progesterone, with our results suggesting E:P may impact fluid regulation at low levels of dehydration (~1% body mass loss). Specifically, we show a possible relationship between lower E:P and copeptin concentration (*P* = 0.054). However, this effect may be more accurately estimated in future research with a larger sample size. Interestingly, there was no relationship observed between E:P and copeptin following greater levels of body mass loss, suggesting that any possible effect of E:P was overridden by the physiological responses to exercise in the heat. Taken together, our data suggest that hormonal fluctuations do not meaningfully alter fluid regulatory responses during exercise in the heat, and that women are not at a particular “disadvantage” during any phase of the menstrual cycle with regard to fluid volume regulation during exercise.

## Data Availability Statement

The raw data supporting the conclusions of this article will be made available by the authors upon reasonable request.

## Ethics Statement

The studies involving human participants were reviewed and approved by University of Connecticut, Institutional Review Board. The patients/participants provided their written informed consent to participate in this study.

## Author Contributions

GG and NC conceived and designed research. GG, MM, and CB performed experiments. ARC analyzed data. GG, ATC, SK, and NC interpreted results of experiments. ARC prepared figures. GG drafted manuscript. GG, NC, MM, CB, ATC, ARC, SK, and DC edited and revised manuscript. All authors approved final version of manuscript.

## Funding

This work was funded in part by NextFlex, Grant/Award Number: PC2.8.

## Author Disclaimer

The views, opinions, and/or findings contained in this article are those of the authors and should not be construed as an official United States Department of the Army position, or decision, unless so designated by other official documentation. Approved for public release, distribution unlimited. Citations of commercial organizations and trade names in this report do not constitute an official Department of the Army endorsement or approval of the products or services of these organizations.

## Conflict of Interest

SK has provided scientific consultation to Danone Research and Quest Diagnostics and has active research grants with Danone Research and Standard Process. DC has served as expert witness and received consulting honoraria from Clif Bar, Sports Innovation Laboratories, and the National Football League, funding from Gatorade, and royalties from Jones and Bartlett, Springer, LWW, Wolters-Kluwer Publishers, Up-to-Date, and Routledge/Taylor & Francis Group. The remaining authors declare that the research was conducted in the absence of any commercial or financial relationships that could be construed as a potential conflict of interest.

## Publisher's Note

All claims expressed in this article are solely those of the authors and do not necessarily represent those of their affiliated organizations, or those of the publisher, the editors and the reviewers. Any product that may be evaluated in this article, or claim that may be made by its manufacturer, is not guaranteed or endorsed by the publisher.
