# Corrigendum: Estrogen to Progesterone Ratio and Fluid Regulatory Responses to Varying Degrees and Methods of Dehydration

**DOI:** 10.3389/fspor.2022.848595

**Published:** 2022-02-10

**Authors:** Gabrielle E. W. Giersch, Nisha Charkoudian, Margaret C. Morrissey, Cody R. Butler, Abigail T. Colburn, Aaron R. Caldwell, Stavros A. Kavouras, Douglas J. Casa

**Affiliations:** ^1^Thermal and Mountain Medicine Division, United States Army Research Institute for Environmental Medicine, Natick, MA, United States; ^2^Oak Ridge Institute for Science and Education, Belcamp, MD, United States; ^3^Korey Stringer Institute, Department of Kinesiology, University of Connecticut, Storrs, CT, United States; ^4^Hydration Science Laboratory, Arizona State University, Tempe, AZ, United States

**Keywords:** female sex hormones, fluid balance, fluid restriction, copeptin, exercise heat stress

In the original article, there were missing citations in the **Introduction** section, paragraph 3, page 2. References Calzone et al. (2001) and Stachenfeld and Taylor (2005) have now been included. The corrected paragraph appears below:

For women, sex hormones interact with the mechanisms governing body fluid balance and circulating levels of fluid regulatory hormones are altered by changes in estrogen and progesterone concentration (Stachenfeld et al., 2000; Calzone et al., 2001; Stachenfeld and Taylor, 2005; Stachenfeld, 2008; Giersch et al., 2019). Estrogen is positively related to AVP and high estrogen concentration decreases the osmotic threshold for AVP synthesis (Stachenfeld et al., 1999; Stachenfeld and Keefe, 2002), suggesting increased AVP secretion at lower levels of dehydration. Estrogen has also been positively correlated with copeptin across the menstrual cycle (Blum et al., 2014). Progesterone has also been observed to impact fluid retention via aldosterone and AVP pathways, and may also increase plasma volume independent of estrogen (Calzone et al., 2001; Stachenfeld and Taylor, 2005), but the precise mechanism of action of progesterone in this regard remains unclear. While estrogen and progesterone both have independent functions throughout the body, they appear to have opposing effects with respect to vascular function (Stephenson and Kolka, 1999; Wenner et al., 2011) and body temperature regulation (Stachenfeld et al., 2001a; Charkoudian and Stachenfeld, 2014). This relationship may also be present with respect to body fluid balance given the varying mechanisms through which estrogen and progesterone alter fluid regulation (Calzone et al., 2001; Stachenfeld et al., 2001b; Stachenfeld and Keefe, 2002; Stachenfeld and Taylor, 2005). Thus, fluid volume regulation may be altered based the concentrations of the hormones relative to each other at a given point in the menstrual cycle (Owen, 1975). This highlights the importance of assessing the relationship between estrogen and progesterone, or the estrogen-to-progesterone ratio (E:P). E:P incorporates the relative concentrations of the two hormones in circulation, allowing for consideration of which is dominant at any given time.

The authors apologize for this error and state that this does not change the scientific conclusions of the article in any way. The original article has been updated.

## Publisher's Note

All claims expressed in this article are solely those of the authors and do not necessarily represent those of their affiliated organizations, or those of the publisher, the editors and the reviewers. Any product that may be evaluated in this article, or claim that may be made by its manufacturer, is not guaranteed or endorsed by the publisher.

## References

[B1] Blum C. A. Mirza U. Christ-Crain M. Mueller B. Schindler C. Puder J. J. (2014). Copeptin levels remain unchanged during the menstrual cycle. PLoS ONE 9:e98240. 10.1371/journal.pone.0098240 24866705 PMC4035325

[B2] Calzone W. L. Silva C. Keefe D. L. Stachenfeld N. S. (2001). Progesterone does not alter osmotic regulation of AVP. Am. J. Physiol. Regul. Integr. Comp. Physiol. 281, R2011–R2020. 10.1152/ajpregu.2001.281.6.R2011 11705788

[B3] Charkoudian N. Stachenfeld N. S. (2014). Reproductive hormone influences on thermoregulation in women. Compr. Physiol. 4, 793–804. 10.1002/cphy.c130029 24715568

[B4] Giersch G. E. W. Charkoudian N. Stearns R. L. Casa D. J. (2019). Fluid balance and hydration considerations for women: review and future directions. Sports Med. 50, 253–261. 10.1007/s40279-019-01206-6 31641955

[B5] Owen J. A. Jr (1975). Physiology of the menstrual cycle. Am. J. Clin. Nutr. 28, 333–338. 10.1093/ajcn/28.4.333 1091131

[B6] Stachenfeld N. S. (2008). Sex hormone effects on body fluid regulation. Exerc. Sport Sci. Rev. 36, 152–159. 10.1097/JES.0b013e31817be928 18580296 PMC2849969

[B7] Stachenfeld N. S. Keefe D. L. (2002). Estrogen effects on osmotic regulation of AVP and fluid balance. Am. J. Physiol. Endocrinol. Metab. 283, E711–E721. 10.1152/ajpendo.00192.2002 12217888

[B8] Stachenfeld N. S. Keefe D. L. Palter S. F. (2001a). Estrogen and progesterone effects on transcapillary fluid dynamics. Am. J. Physiol. Regul. Integr. Comp. Physiol. 281, R1319–R1329. 10.1152/ajpregu.2001.281.4.R1319 11557642

[B9] Stachenfeld N. S. Silva C. Keefe D. L. (2000). Estrogen modifies the temperature effects of progesterone. J. Appl. Physiol. (1985) 88, 1643–1649. 10.1152/jappl.2000.88.5.1643 10797125

[B10] Stachenfeld N. S. Silva C. Keefe D. L. Kokoszka C. A. Nadel E. R. (1999). Effects of oral contraceptives on body fluid regulation. J. Appl. Physiol. (1985) 87, 1016–1025. 10.1152/jappl.1999.87.3.1016 10484572

[B11] Stachenfeld N. S. Splenser A. E. Calzone W. L. Taylor M. P. Keefe D. L. (2001b). Selected contribution: sex differences in osmotic regulation of AVP and renal sodium handling. J. Appl. Physiol. 91, 1893–1901. 10.1152/jappl.2001.91.4.1893 11568177

[B12] Stachenfeld N. S. Taylor H. S. (2005). Progesterone increases plasma volume independent of estradiol. J. Appl. Physiol. (1985) 98, 1991–1997. 10.1152/japplphysiol.00031.2005 15718411

[B13] Stephenson L. A. Kolka M. A. (1999). Esophageal temperature threshold for sweating decreases before ovulation in premenopausal women. J. Appl. Physiol. 86, 22–28. 10.1152/jappl.1999.86.1.22 9887109

[B14] Wenner M. M. Taylor H. S. Stachenfeld N. S. (2011). Progesterone enhances adrenergic control of skin blood flow in women with high but not low orthostatic tolerance. J. Physiol. 589, 975–986. 10.1113/jphysiol.2010.194563 21173076 PMC3060374

